# Manganese Neurotoxicity as a Complication of Chronic Total Parenteral Nutrition

**DOI:** 10.1155/2020/9484028

**Published:** 2020-04-23

**Authors:** Alisha Khan, Jonathan Hingre, Amit S. Dhamoon

**Affiliations:** ^1^Department of Medicine, SUNY Upstate Medical University, Syracuse, NY, USA; ^2^College of Medicine, SUNY Upstate Medical University, Syracuse, NY, USA

## Abstract

Manganese accumulation in the central nervous system creates clinical symptoms of cognitive dysfunction, behavioral changes, and movement disorders resembling Parkinson's disease. Radiographic features of this rare clinical entity include symmetric T1 hyperintensities in the bilateral globus pallidi, with corresponding hypointensities on T2-weighted images. Total parenteral nutrition (TPN) is an increasingly used potentially lifesaving therapy for patients who cannot tolerate enteral nutrition. However, when used over a period of several weeks to months, its associated risks and complications carry significant morbidity and mortality. One of the more rare complications of TPN use is manganese toxicity. We provided care for a 38-year-old female on chronic TPN who presented to the hospital with Parkinsonian features, confusion, falls, and lethargy. MRI brain showed T1 hyperintensities in the bilateral globus pallidi, which were attributed to manganese toxicity from chronic TPN use. Supporting evidence for this rare entity included decreased signal intensity in the bilateral globus pallidi on T2-weighted images and T1 hyperintensities in the substantia nigra. With antifungal treatment and permanent cessation of TPN, her mentation and neurological symptoms began to improve within a week. Repeat MRI brain performed one month after discontinuation of TPN revealed improvement of the T1 hyperintensities in the bilateral globus pallidi. Our objective in presenting this case is to highlight manganese neurotoxicity as a rare complication of TPN in a patient without known hepatic dysfunction and to emphasize the importance of routinely monitoring patients for the possible adverse effects of chronic TPN. Our case is among the handful of published cases in which a patient without known liver dysfunction, which is the primary organ responsible for manganese elimination from the body, developed manganese neurotoxicity.

## 1. Introduction

Total parenteral nutrition (TPN) is a short-term potentially lifesaving therapy for patients who cannot tolerate enteral nutrition. TPN also has several risks and patients need to be monitored closely while on therapy. One of the more rare complications of TPN is manganese toxicity. Manganese is an essential dietary mineral tightly regulated in humans through the control of absorption, distribution, storage, and excretion. Elevated tissue levels of manganese can occur from excessive exposure, often through inhalation or parenteral exposures [1]. Parenteral exposure to manganese has high bioavailability because normal regulatory mechanisms in the gastrointestinal tract are bypassed. Manganese is cleared through the liver, increasing the risk for overload in patients with hepatic dysfunction [2]. Manganese accumulation in the central nervous system creates clinical symptoms of cognitive dysfunction, behavioral changes, and movement disorders resembling Parkinson's disease. Clinical features include bradykinesia, tremor, weakness, muscle rigidity, mask-like facies, postural instability, gait disturbances, headaches, confusion, and somnolence [3]. In this report, we will further describe the clinical presentation and corresponding imaging results of manganese toxicity due to TPN.

## 2. Case Presentation

Our patient is a 38-year-old woman with a past medical history most significant for a Roux-en-Y gastric bypass surgery in March 2017, complicated by a marginal ulcer at the anastomosis site with subsequent perforation. The patient could not tolerate oral nutrition and became severely malnourished, and a PICC line was placed for TPN in December 2017. Chronic TPN use led to recurrent candidemia with blood cultures growing *Candida albicans* resistant to fluconazole and voriconazole. Over several months, her PICC line was replaced thrice, and she was ultimately treated with micafungin. The patient had multiple admissions over the past few months for workup of altered mental status and unsteady gait. Her other past medical history included systemic lupus erythematosus treated with mycophenolate mofetil and hydroxychloroquine, left portal vein branch thrombosis on enoxaparin therapy, type 2 diabetes mellitus not on insulin, fibromyalgia, depression, and anxiety. She presented to our hospital in July 2018 with complaints of tremors, impaired mobility, confusion, gait instability, falls, and lethargy.

On admission, she was noted to be confused, with horizontal nystagmus and dilated pupils as well as bradypnea. Dilated fundus examination was indicative of candida retinitis. She was lethargic but arousable. Her speech was difficult to understand, and the history of presenting illness was provided primarily by the patient's mother who relayed to the team that she had been having tremors, episodes of confusion, and numerous falls for the past several months. Her mother also noticed that she had increased irritability and emotional lability during this time. She was initially admitted to the intensive care unit for airway management due to her acute encephalopathy. Due to a lack of meningeal signs, a lumbar puncture was not performed.

**Figure 1:**
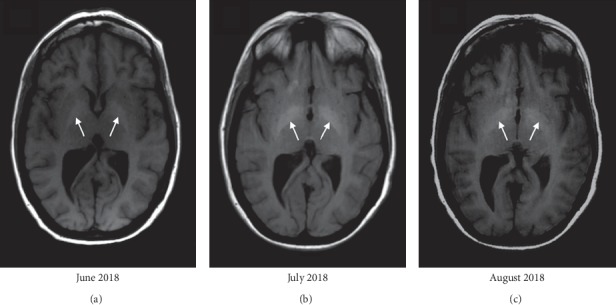
Axial T1-weighted MRI brain performed serially over three months, showing the progression of the T1 hyperintensities in the globus pallidi from June 2018 to July 2018, followed by the improvement of the T1 hyperintensities in August 2018, one month after discontinuation of TPN. (a) Evidence of developing T1 hyperintensities in the bilateral globus pallidi on a previous hospital admission in June 2018. (b) Further increase in signal in the bilateral globus pallidi on T1-weighted images in July 2018, corresponding to her pronounced Parkinsonian features on clinical presentation. (c) Improvement in the T1 hyperintensities in the globus pallidi, collected one month after cessation of TPN use and corresponding to near-resolution of her Parkinsonism.

**Figure 2:**
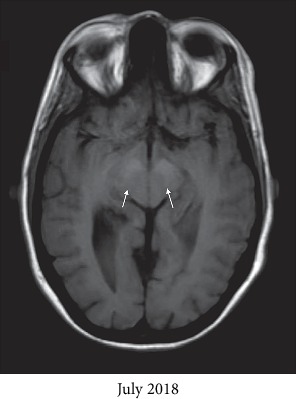
Axial T1-weighted MRI brain from July 2018 showing hyperintensities in the substantia nigra, as evidenced by the two white arrows.

**Figure 3:**
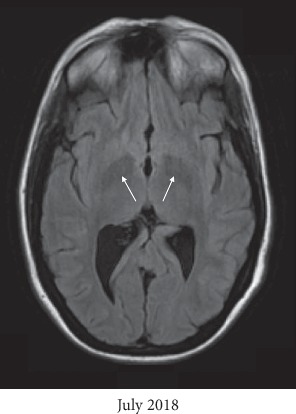
Supporting evidence for manganese neurotoxicity includes decreased signal intensity in the bilateral globus pallidi on axial T2-weighted images of the MRI brain from July 2018.

The infectious disease service was consulted, and she was started on liposomal amphotericin B and flucytosine for a total of 6 weeks. MRI brain showed worsening T1 hyperintensities from June 2018 to July 2018 in the bilateral globus pallidi, as evidenced by the arrows in Figures 1(a) and 1(b), which were thought to be secondary to manganese toxicity from chronic TPN use. Furthermore, a repeat MRI brain was performed in August 2018, one month after discontinuation of TPN, which showed improvement of the T1 hyperintensities in the bilateral globus pallidi, as evidenced by the white arrows in Figure 1(c), approaching the intensity level noted in June 2018. Other supporting findings included T1 hyperintensities in the substantia nigra, as evidenced by the arrows in Figure 2, and decreased signal in the bilateral globus pallidi on T2-weighted images, as evidenced by the arrows in Figure 3. With antifungal treatment and cessation of TPN, her mentation and neurological symptoms improved within 3-4 days of cessation of TPN.

General surgery was consulted for gastrostomy tube placement in her remnant stomach, as the patient was unable to consume more than 500 calories by mouth per day due to complaints of nausea and vomiting. Although our patient presented to the hospital seven months after initiation of TPN therapy, her Parkinsonian symptoms had begun to manifest after three months on TPN.

## 3. Investigations

Her MRI brain, shown in Figure 1, showed symmetric T1 hyperintensities in the bilateral globus pallidi, a finding compatible with manganese toxicity. A serum manganese (Mn) level was elevated at 4.0 *μ*g/L (reference range <2.5 *μ*g/L), collected 1 day after TPN was permanently discontinued. Relevant laboratory investigations revealed a copper level of 22 *μ*g/dL, zinc level of 44 *μ*g/dL, vitamin B1 level of 149.8 nmol/L, vitamin B12 level of 703 ng/L, and 1,25-dihydroxyvitamin D level of 7.3 ng/mL, for which she was started on weekly supplementation. CBC on admission showed a WBC count of 7.5 × 10^3^/*μ*L, haemoglobin level of 9.2 g/dL, haematocrit level of 27.6%, platelet level of 453 × 10^3^/*μ*L, MCV of 79.9 fL, and red cell distribution width of 18.2%. CMP on admission showed the following levels: sodium 146 mmol/L, potassium 4.8 mmol/L, chloride 107 mmol/L, bicarbonate 26 mmol/L, blood urea nitrogen 27 mg/dL, creatinine 0.94 mg/dL, glucose 228 mg/dL, calcium 8.5 mg/dL, ALT 40 U/L, AST 28 U/L, albumin 3.3 g/dL, alkaline phosphatase 159 IU/L, anion gap 13 mEq/L, total bilirubin <0.2 mg/dL, direct bilirubin <0.1 mg/dL, and osmolality 314 mmol/kg.

The composition of the TPN solution she received from December 2017 to July 2018 was as follows: 40 g of 15% amino acids solution, 120 g of 70% dextrose solution, 15 g of 30% fat emulsion, 21.1 g of 4 mEq/mL sodium chloride solution, 10 mmol of 45 mmol/15 mL sodium phosphate solution, 45.5 mEq of 4 mEq/mL sodium acetate solution, 2.15 g of 10% calcium gluconate solution, 70 mEq of 2 mEq/mL potassium chloride solution, 2.22 g of 50% magnesium sulfate solution, 10 mL of adult multivitamin solution, 100 mg of 100 mg/mL thiamine solution, and 978.96 mL of sterile water solution. Importantly, a 1 mL injection of a solution containing trace elements (Cr-Cu-Mn-Se-Zn: 10-1000-500-60 mcg/mL) was added to each bag of TPN. In other words, each bag of TPN contained 500 mcg of manganese.

## 4. Differential Diagnosis

- T1 hyperintensities in the globus pallidus and clinical features of Parkinson's have been noted in individuals with inherited mutations of SLC30A10, a Mn transporter gene.
- Bilateral pallidal hyperintensity can also be seen in patients with ischemic or hypoxic encephalopathy, hypoparathyroidism or pseudohypoparathyroidism, hamartomas of neurofibromatosis type 1, and cerebral haemorrhage due to Japanese encephalitis; however, these conditions would not present with Parkinsonism.
- Ephedrone abuse is an emerging cause of Mn-induced Parkinsonism.
- Both inherited and acquired hypermanganesemia have similar presentations and lead to Mn deposition in the basal ganglia and hyperintense signalling on T1-weighted images.
- Prior to brain imaging, infective causes of her presentation would have included encephalitis, fungemia, and bacteremia; however, these were less likely as the patient was afebrile and did not have an abnormal white cell count or localizing neurological signs. Noninfective causes would include drug intoxication, aseptic meningitis, and autoimmune meningitis with her history of SLE.

## 5. Discussion

Manganese (Mn) is a metal that is essential for the human body. It is a requirement for the maintenance of the nervous and immunological systems, acts as a cofactor for many enzymes, and participates in the regulation of blood sugar levels and vitamin metabolism [4–7]. In particular, Mn is a required component of Mn metalloproteins, which function as oxidoreductases, transferases, hydrolases, lyases, isomerases, and ligases [8]. Mn is also required for the functioning of enzymes such as arginase, glutamine synthetase, phosphoenolpyruvate decarboxylase, pyruvate carboxylase, and Mn superoxide dismutase [9]. These are some of the reasons why Mn is a component of nutritional support formulas such as TPN, as well as infant and neonatal formulas. Mn has a propensity to form tight complexes with other molecules normally leading to low free plasma and tissue concentrations [10, 11].

A metal's concentration in various parts of the body depends on its absorption, distribution, storage, and excretion. Despite the fact that there are over a dozen proteins involved in maintaining Mn homeostasis, including the divalent metal transporter, transferrin and transferrin receptor, superoxide dismutase, ceruloplasmin, and ferroportin [12], among various others, manganese toxicity develops relatively rapidly due to the mechanisms by which our bodies regulate Mn as discussed below.

### 5.1. Absorption

Enteric absorption in the small intestine and absorption from the pulmonary system following airborne, usually occupational exposure, are the most common ways through which Mn enters our bodies [4]. Water contaminated with Mn from nonpublic sources can also lead to toxicity [13]. Inhaled Mn can bypass first-pass metabolism by the liver as well as the blood-brain barrier to enter the nervous system via the olfactory tract [4, 14, 15]. Intravenous injection of illicit narcotics is an emerging cause of Mn toxicity [16]. A less common mechanism for manganese toxicity is prolonged TPN use, as well as prolonged use of infant and neonatal formulas.

### 5.2. Distribution and Storage

Mn accumulates mainly in the liver, brain, and bone; however, the brain is the organ most likely to be affected by Mn toxicity. It is postulated that the choroid plexus transports Mn into the nervous system, as Mn is detectable in the cerebrospinal fluid before it is detected in the brain tissue [17]. Mn can also enter the brain through the capillary endothelial cells of the blood-brain barrier or directly by the way of the olfactory nerve [7]. In rat brains, the globus pallidi, followed by the substantia nigra pars compacta, thalamus, caudate putamen, axon bundles, and cortex, accumulate the highest concentrations of Mn [4, 18]. In humans, the superior and inferior colliculi, amygdala, stria terminalis, and hippocampus are additional susceptible areas [4]. In these areas, the half-life of Mn is 5–7 days, which is longer than in other organs [19]. In rat models, even when Mn reaches a steady-state concentration in the blood, the CSF concentration of Mn continues to rise due to its slower elimination rate, which likely contributes to the fact that the brain is the organ most susceptible to Mn toxicity [4, 20]. In addition, iron deficiency can also precipitate Mn toxicity, as both Mn and Fe compete for similar transport proteins [21].

### 5.3. Excretion

The fecal hepatobiliary system is the primary route of Mn elimination, accounting for 80% of Mn elimination, followed by the urinary system [4, 22]. Sweat and breast milk also serve as the minor routes of elimination [4, 23]. Therefore, in individuals with liver failure, due to the decreased excretion of Mn in bile, Mn toxicity becomes more likely. Mn hepatic encephalopathy is a term that has been coined to specifically identify individuals in whom hepatic dysfunction is the primary cause of Mn neurotoxicity.

### 5.4. Manganese Toxicity

Although idiopathic Parkinson's disease and Mn-induced neurotoxicity both present with extrapyramidal symptoms, their target areas in the brain differ, resulting in similar but differing clinical presentations. Mn primarily affects the globus pallidus and the striatum of the basal ganglia, whereas idiopathic Parkinson's disease affects the substantia nigra [7]. Mn preferentially deposits in the dopaminergic cells of the basal ganglia, notably the globus pallidus, resulting in extrapyramidal motor disturbances presenting similarly to Parkinson's disease. In fact, Mn has been found to upregulate histone deacetylase (HDAC) and downregulate histone acetyltransferase (HAT) in neuronal cells, which damages dopaminergic neurons [24].

Mn has been found to disrupt the signalling of dopamine, serotonin, and glutamine, as evidenced by synchrotron X-ray fluorescent imaging, which provides insight into Mn toxic kinetics and its neural distribution. High levels of Mn exposure have also been noted to decrease the copper levels in the subventricular and subgranular zones of the brain, which have been postulated to be responsible for the nonmotor symptoms of Mn-induced Parkinsonism [4, 25, 26].

Our patient's TPN solution contained 500 mcg of Mn, which we would argue is an excessive amount, even though many commercial TPN formulas contain up to 800 mcg of Mn. Takagi et al. performed a study in which patients received 0, 55, 110, and 1100 mcg of Mn/day using an on/off design [27]. Elevated blood Mn levels were noted when patients received 110 or 1100 mcg of Mn/day, but not when receiving 55 mcg of Mn/day [27]. Takagi et al., therefore, concluded that the recommended dose of Mn for stable patients receiving TPN at home is 55 mcg/day [27]. As a result, the American Society for Parenteral and Enteral Nutrition (ASPEN) has adopted the recommendation that parenteral nutrition formulas should contain 40 to 100 mcg of Mn/day, with a daily requirement set at 55 mcg/day.

### 5.5. Treatment

Treatment of Mn toxicity is dependent on eliminating the source of Mn exposure. However, methods such as chelation with intravenous EDTA, which increase urinary excretion of Mn, do not seem to improve the symptoms of Mn toxicity [4, 7, 26] but can still be used in an emergency setting to decrease the blood concentration of Mn [4]. For this reason, in the case of our patient, chelation therapy was not attempted. In addition, her symptoms improved within a few days of TPN discontinuation, which is the cornerstone of treatment. Of note, in cases of normal serum or urine manganese levels, noting an abnormally elevated urinary excretion of manganese following chelation therapy can also be indicative of manganese toxicity. When chelation therapy is used in conjunction with iron supplementation, however, a significant improvement of neurological symptoms can be expected [28].

Following Mn toxicity, internal organs themselves, particularly bones which house 40% of the body's total Mn stores [29] and where Mn has been found to have a half-life of 8-9 years, serve as a continuous internal source of Mn exposure [4]. However, cases have been described in the literature in which the characteristic MRI findings and clinical Parkinsonian symptoms have resolved following elimination of the Mn source or after hepatic transplantation [30, 31]. As the liver is the organ primarily responsible for the elimination of Mn, liver dysfunction can precipitate Mn toxicity. Liver transplantation has thus been shown to reduce the Mn burden on the brain on repeat T1-weighted MRI imaging performed months after the surgery [32].

In the past, it was hypothesized that treatment with levodopa may improve the extrapyramidal symptoms of Mn-induced neurotoxicity; however, response to levodopa is short-lived, lasting only two to three years, and even ten years after eliminating the source of Mn, the severity of patients' symptoms continued to progress [7, 33, 34].

## 6. Conclusion

Total parenteral nutrition should not be used as a chronic source of nutrition. Fungemia, fungal retinitis, and manganese toxicity are complications of TPN that may develop with long-term therapy. Parkinsonian features of manganese toxicity were visible in our patient approximately three months after initiation of TPN although the time frame can be highly variable depending on individual patient characteristics, particularly liver function and individual pharmacokinetic mechanisms regulating manganese absorption, distribution, storage, and excretion. One may expect the Parkinsonian features of manganese toxicity to develop even sooner than three months in patients with liver dysfunction. Current guidelines recommend monitoring patients for manganese toxicity if TPN is administered for greater than 30 days. In addition, being mindful of the fact that the recommended daily dose of Mn in TPN formulations is 55 mcg/day is essential, as many TPN formulations contain more than the recommended amount. Both providers and patients should be vigilant and educated about monitoring for complications of TPN, especially in patients with liver disorders.
